# Factors Associated With Cancer Worry Among People Aged 50 or Older, Spain, 2012–2014

**DOI:** 10.5888/pcd12.150398

**Published:** 2015-12-24

**Authors:** Jesús López-Torres Hidalgo, Joseba Rabanales Sotos, María José Simarro Herráez, Monchi Campos Rosa, Jaime López-Torres López, María Pilar Sánchez Ortiz

**Affiliations:** Author Affiliations: Joseba Rabanales Sotos, Cuenca Faculty of Nursing, University of Castile-La Mancha, Albacete; María José Simarro Herráez, Health Service of Castile-La Mancha, Villarrobledo Health Centre, Albacete; Monchi Campos Rosa, Albacete University Teaching Hospital Complex, Albacete; Jaime López-Torres López, Valencia University Teaching Hospital and La Fe Polytechnic Institute, Valencia; María Pilar Sánchez Ortiz, Health Center 2 Hellín, Hellín, Albacete, Castile-La Mancha Health Service, Albacete. Dr López-Torres Hidalgo is also affiliated with Albacete Zone IV Health Center, Castile-La Mancha Health Service, and Albacete Faculty of Medicine, University of Castile-La Mancha, Albacete.

## Abstract

**Introduction:**

Cancer worry varies among patients and may influence their participation in preventive activities. We tested whether sociodemographic characteristics, lifestyle, locus of control, comorbidity, and perceived health status were associated with the level of cancer worry among adults aged 50 or older.

**Methods:**

We conducted an observational cross-sectional study of 666 adults in Spain aged 50 or older. Participants were selected by simple random sampling and asked to visit their designated health center for a personal interview. The study variables were level of cancer worry (measured by Cancer Worry Scale [CWS]), sociodemographic characteristics, lifestyle, personal history or family history of cancer, comorbidity, self-perceived health, locus of control, and social support.

**Results:**

More than half of participants, 58.1%, were women; mean age was 60.5 years (standard deviation [SD], 6.8 y). Measurement of the frequency and severity of cancer worry (possible scale of 6–24 points) yielded a mean CWS score of 9.3 (95% confidence interval, 9.0–9.5); 31.9% of participants reported being concerned about cancer. Scores were higher among women (9.7 [SD, 3.3]) than men (8.7 [SD, 2.7]) (*P* < .001) and among participants in rural settings (10.0 [SD, 3.4]) than in urban settings (9.0 [SD, 3.0]) (*P* < .001). Multiple linear regression showed a greater degree of cancer worry among people with personal or family history of cancer, more health problems, worse self-perceived health, and lower social support.

**Conclusion:**

Cancer worry is frequent among older adults, and the level of such concern is related not only to personal characteristics but also to lifestyle and health status. Further research is required to understand how contextual factors can influence cancer worry and how such concern changes behavior patterns related to cancer prevention activities.

## Introduction

“Worry is a cognitive activity wherein an individual experiences a series of negative thoughts about an uncertain issue” (1). During a single lifetime, health and disease alternate and, depending on the environmental circumstances and genetic characteristics, proneness to disease varies significantly from one person to another. However, the possibility of falling ill — particularly with potentially severe diseases such as cancer —tends to worry everybody. To some people, the possibility of falling ill is a constant threat and can give rise to a high level of emotional stress, which can interfere with social and family life.

In Spain, cancer is diagnosed in one of every 3 men and one of every 5 women (2). The Spanish Society of Medical Oncology (Sociedad Española de Oncología Médica) indicated that 79% of the Spanish population reported cancer as the disease about which they worried most, followed by acquired immunodeficiency syndrome (AIDS) (34%) and Alzheimer’s disease (24%) (2). In general, cancer arouses worry and anxiety, and concern about having it can vary according to personal characteristics, such as sex or socioeconomic level, although little is known about which population groups are most worried (3). Worry is a key element in health behavior and attitudes toward preventive health care (1) and participation in cancer screening programs. Studies have yielded contradictory results; it is not clear whether negative emotions, such as worry, favor or hinder a person’s participation in preventive activities (4–6). Preventive activities are particularly important from the age of 50 on, because the risk of some types of cancer, such as breast and colorectal cancers, increases at this age, and cancer screening is recommended. Cancer worry levels that could be considered disproportionate are associated with unsatisfactory levels of adherence to prevention programs (2). It is therefore important for health professionals to explore cancer worry levels with their patients to identify those who show intense concern and, as a result, refuse to be screened for cancer. In such patients, appropriate information about the real possibilities of cancer and the usefulness of preventive activities could reduce their level of worry.

Cancer worry is a complex phenomenon which, though not well understood, might be related to an individual’s personal characteristics, health status, or lifestyle. It would be desirable to identify physical, mental, and social variables that could account for the different levels of cancer worry among people. The aim of this study was to describe cancer worry levels among adults aged 50 or over, according to their sociodemographic characteristics, lifestyle, and other factors.

## Methods

We conducted an observational cross-sectional study of adults aged 50 or older who reside in 8 basic health areas in the Province of Albacete in southwest Spain.

### Recruitment

The initially envisaged sample size was 652 participants, calculated on the basis of a standard deviation (SD) in Cancer Worry Scale (CWS) scores of 2.8 points, a 95% confidence level, and a precision of ±0.2 points. The sample size was increased by 30% to offset nonresponses; the final size was 848. The following exclusion criteria were applied: sensory deficits incompatible with participating in a personal interview; immobility; or moderate or severe cognitive impairment or low intellectual performance.

Through simple random sampling, potential participants were selected on the basis of health card data, which reflect all people entitled to health care under Spain’s National Health System. Of the 848 people selected, 666 (78.5%) agreed to participate, subsequently attended their health center on the appointed date, and were interviewed by health care staff; 153 (18.0%) refused to participate or did not keep the interview appointment, and 29 (3.4%) were unable to participate for various reasons (impossible to locate, severe disease, or death). Interviews lasted for 30 to 45 minutes. Data were collected from October 2012 through September 2014. The study was approved by the Clinical Research Ethics Committee of the Albacete Health Area.

### Measures

Level of cancer worry was measured by the Spanish version of the CWS (2), consisting of 6 questions with 4 response options (1 = not at all or rarely; 2 = sometimes; 3 = often; 4 = almost all the time), such that each individual attains a score ranging from 6 (minimum worry) to 24 (maximum worry). The CWS assesses the frequency with which a person worries about cancer, the impact of worry on mood and daily activities, concern about developing cancer, and the importance of cancer worry to the person. Although numerous instruments assess cancer worry (7–9), one of the most widely used is the CWS, developed by Lerman et al in 1991 (10), initially validated in an English population by Hopwood et al in 2001 (11), and subsequently validated in a Spanish population by Cabrera et al in 2011 (2). The CWS can be used in both primary and specialized care to identify people with high levels of cancer worry. Not only does it provide a numerical value that measures degree of worry but the CWS also furnishes descriptive information for ascertaining the individual’s degree of worry at different points in time, thus addressing the 2 fundamental dimensions of cancer worry, ie, severity and frequency (6).

Sociodemographic characteristics included age, place of residence (urban or rural setting), educational level, marital status, form of cohabitation, and occupation-based social class.

Lifestyle factors included the following: level of physical activity, measured by the International Physical Activity Questionnaire (12); smoking habits; alcohol consumption (with hazardous drinking defined as consuming 280 g of alcohol per week for men and 168 g for women); and type of diet, assessed by a food frequency questionnaire (Cuestionario de Frecuencia de Consumo de Alimentos) (13) in accordance with guidelines of the Spanish Society of Community Nutrition (Sociedad Española de Nutrición Comunitaria).

For each study participant, we also ascertained information on personal history of cancer and history of cancer among first-degree relatives; number of reported health problems; comorbidity (by using the corrected Charlson Index [14]); self-perceived health, classified as very poor, poor, fair, good, or very good; functional status, assessed with the EuroQol-5D questionnaire (15), which includes items on mobility, personal care, daily activities, pain or discomfort, and anxiety or depression; locus of control (internal, external, random, or indeterminate), assessed using the 9-item Font scale (16); and social support, assessed by the 11-item Duke–UNC Functional Social Support Questionnaire (17), which uses a Likert-type response scale of 1 to 5 for each question and has a score range from 11 to 55 points.

### Analyses

Participants’ responses were entered into a database, processed, and analyzed. We analyzed participants descriptively, using distribution of frequencies or measures of central trend and dispersion, depending on the variable. We checked for associations using tests of comparison of means (*t* test and analysis of variance [ANOVA], with multiple post-hoc comparisons by the Scheffé test). Using the CWS score as the dependent variable, a multiple linear regression model was constructed for explanatory purposes by the stepwise method, to ascertain related variables and avoid possible confounding factors. The following independent variables were included in the model: level of physical activity, self-perceived health, personal and family history of cancer, social support, number of reported health problems, locus of control, and sociodemographic characteristics that had a significant association (*P* < .05). We introduced age, number of health problems, and Duke–UNC scale scores as quantitative variables and the remainder as dichotomous variables. Coefficients were estimated using the least squares or maximum likelihood method, and the independence of the residual values was checked with the Durbin–Watson test. To identify variables associated with higher cancer worry levels (score of >10 points on the CWS, equivalent to the 66th percentile of distribution), a logistic regression model was fitted using the stepwise procedure. All statistical analyses were performed using SPSS version 19.0 (IBM Corporation).

## Results

Of 666 participants, 279 were men (41.9%) and 387 were women (58.1%); mean age was 60.5 (SD, 6.8) years (Table 1); 51.2% reported moderate levels of physical activity, 80.2% were nonsmokers, and 74.5% rated their health as good or very good (Table 2). Table 3 shows the characteristics of the participants by tertiles of CWS.

**Table 1:** Sociodemographic Characteristics of Participants (N = 666) in Study on Cancer Worry Among Adults Aged 50 or Older, Spain, 2012–2014^a^

Characteristic	Men, n (%)	Women, n (%)	Total, n (%)
**Age, y**			
50–64	194 (69.5)	271 (70.0)	465 (69.8)
≥65	85 (30.5)	116 (30.0)	201 (30.2)
**Rural or urban setting**			
Rural	79 (28.3)	104 (26.9)	183 (27.5)
Urban	200 (71.7)	283 (73.1)	483 (72.5)
**Educational level**			
No formal education	45 (16.1)	79 (20.4)	124 (18.7)
Primary	133 (47.7)	192 (49.6)	325 (48.8)
Secondary	57 (20.4)	66 (17.1)	123 (18.5)
University	44 (15.8)	50 (12.9)	94 (14.1)
**Marital status**			
Single	17 (6.1)	32 (8.3)	49 (7.4)
Married or stable union	239 (85.7)	293 (75.7)	532 (79.9)
Divorced	19 (6.8)	28 (7.2)	47 (7.1)
Widowed	4 (1.4)	34 (8.8)	38 (5.7)
**Form of cohabitation**			
Lives alone	20 (7.2)	37 (9.6)	57 (8.6)
Lives with spouse (with or without children)	246 (88.2)	287 (74.8)	533 (80.4)
Other forms of cohabitation	13 (4.6)	60 (15.6)	73 (11.0)
**Social class**			
Classes I and II: higher-grade and lower-grade professionals, administrators and officials, managers, professions linked to university degrees	36 (13.0)	53 (13.7)	89 (13.4)
Classes III and IV: middle management and managerial staff, self-employed persons	58 (20.8)	43 (11.1)	101 (15.2)
Class V: lower-grade skilled technicians	89 (31.9)	135 (34.9)	224 (33.6)
Classes VI and VII: semi-skilled and unskilled workers	96 (34.4)	156 (40.3)	252 (37.9)

a Through simple random sampling, participants residing in 8 basic health areas in the Province of Albacete in southwest Spain were selected on the basis of health card data, which reflect all people entitled to health care under Spain’s National Health System.

**Table 2:** Lifestyle and Health Characteristics of Participants (N = 666) in Study on Cancer Worry Among Adults Aged 50 or Older, Spain, 2012–2014^a^

Characteristic	Men, n (%)	Women, n (%)	Total, n (%)
**Level of physical activity^b^**			
High	44 (15.8)	44 (11.4)	88 (13.2)
Moderate	138 (49.5)	203 (52.5)	341 (51.2)
Low or inactive	97 (34.8)	140 (36.2)	237 (35.6)
**Smoking habit**			
Yes	70 (25.1)	62 (16.0)	132 (19.8)
No	209 (74.9)	325 (84.0)	534 (80.2)
**Alcohol consumption (hazardous drinker)^c^**			
Yes	15 (5.4)	7 (1.8)	22 (3.3)
No	264 (94.6)	380 (98.2)	644 (96.7)
**Balanced diet^d^**			
Yes	91 (32.6)	157 (40.6)	248 (37.2)
No	187 (67.0)	228 (58.9)	415 (62.3)
Did not respond to question	1 (0.4)	2 (0.5)	3 (0.5)
**Personal history of cancer**			
Yes	29 (10.4)	29 (7.5)	58 (8.7)
No	250 (89.6)	358 (92.5)	608 (91.3)
**Family history of cancer**			
Yes	118 (42.3)	204 (52.7)	322 (48.3)
No	161 (57.7)	183 (47.3)	344 (51.7)
**Number of health problems**			
None	60 (21.5)	64 (16.5)	124 (18.6)
1 or 2	137 (49.1)	216 (55.8)	353 (53.0)
≥3	82 (29.4)	107 (27.6)	189 (28.4)
**Charlson Comorbidity Index^e^**			
0 or 1 point	106 (38.0)	178 (46.0)	284 (42.6)
2 points	105 (37.6)	123 (31.8)	228 (34.2)
≥3 points	68 (24.4)	86 (22.2)	154 (23.1)
**Self-perceived health**			
Very poor or poor	6 (2.2)	15 (3.9)	21 (3.2)
Fair	47 (16.8)	102 (26.4)	149 (22.4)
Good or very good	226 (81.0)	270 (69.8)	496 (74.5)
**Functional limitation^f^**			
Yes	48 (17.2)	104 (26.9)	152 (22.8)
No	231 (82.8)	283 (73.1)	514 (77.2)
**Locus of control^g^**			
Internal	188 (67.4)	249 (64.3)	437 (65.6)
External	41 (14.7)	65 (16.8)	106 (15.9)
Random	11 83.9)	26 (6.7)	37 (5.6)
Indeterminate	37 (13.3)	42 (10.9)	79 (11.9)
Did not respond to question	2 (0.7)	5 (1.3)	7 (1.1)
**Social support^h^**			
Weak	11 (3.9)	20 (5.2)	31 (4.7)
Normal	265 (95.0)	361 (93.3)	626 (94.0)
Did not respond to question	3 (1.1)	6 (1.6)	9 (1.4)

a Through simple random sampling, participants residing in 8 basic health areas in the Province of Albacete in southwest Spain were selected on the basis of health card data, which reflect all people entitled to health care under Spain’s National Health System.

b Measured by International Physical Activity Questionnaire (12).

c Defined as consuming 280 g of alcohol per week (men) and 168 g per week (women).

d Assessed by a food frequency questionnaire (Cuestionario de Frecuencia de Consumo de Alimentos) (13) in accordance with guidelines of the Spanish Society of Community Nutrition (Sociedad Española de Nutrición Comunitaria).

e Charlson et al (14).

f Assessed with the EuroQol-5D questionnaire (15), which includes items on mobility, personal care, daily activities, pain or discomfort, and anxiety or depression.

g Refers to the extent to which people believe outcomes are determined by what they do (internal control) or are determined by events outside their control (external control); assessed using the 9-item Font scale (16).

h Assessed by the 11-item Duke–UNC Functional Social Support Questionnaire (17).

**Table 3:** Sociodemographic, Lifestyle, and Health Characteristics of Participants (N = 666) in Study on Cancer Worry Among Adults Aged 50 or Older, by Tertiles of Cancer Worry Scale (CWS)^a^ Scores, Spain, 2012–2014^b^

Characteristic	CWS Score			*P* Value for Linear Trend
	1st tertile (≤7 Points), n (%)	2nd Tertile (8–10 Points), n (%)	3rd Tertile (>10 Points), n (%)	
**Age, y**				
50– 64	153 (65.4)	162 (70.4)	150 (74.3)	.04
≥65	81 (34.6)	68 (29.6)	52 (25.7)	
**Rural or urban setting**				
Rural	49 (20.9)	63 (27.4)	71 (35.1)	.001
Urban	185 (79.1)	167 (72.6)	131 (64.9)	
**Educational level**				
No formal education or primary only	161 (68.8)	141 (61.3)	147 (72.8)	.44
Secondary or university	73 (31.2)	89 (38.79	55 (27.2)	
**Marital status**				
Single, divorced, or widowed	51 (21.8)	50 (21.7)	33 (16.3)	.17
Married or stable union	183 (78.2)	180 (78.3)	169 (83.7)	
**Form of cohabitation**				
Living alone	23 (9.8)	22 (9.6)	12 (6.0)	.17
Living with spouse or other form of cohabitation	211 (90.2)	208 (90.4)	187 (94.0)	
**Social class^c^**				
Classes I–IV	63 (26.9)	74 (32.2)	53 (26.2)	.93
Classes V–VII	171 (73.1)	156 (67.8)	149 (73.8)	
**Level of physical activity^d^**				
High or moderate	135 (57.7)	155 (67.4)	139 (68.8)	.01
Low or inactive	99 (42.3)	75 (32.6)	63 (31.2)	
**Smoking habit**				
Yes	42 (17.9)	46 (20.0)	44 (21.8)	.32
No	192 (82.1)	184 (80.0)	158 (78.2)	
**Alcohol consumption (hazardous drinker)^e^**				
Yes	8 (3.4)	8 (3.5)	6 (3.0)	.80
No	226 (96.6)	222 (96.5)	202 (97.0)	
**Balanced diet^f^**				
Yes	89 (38.0)	88 (38.3)	71 (35.7)	.62
No	145 (62.0)	142 (61.7)	128 (64.3)	
**Personal history of cancer**				
Yes	14 (6.0)	18 (7.8)	26 (12.9)	.01
No	220 (94.0)	212 (92.2)	176 (87.1)	
**Family history of cancer**				
Yes	102 (43.6)	108 (47.0)	112 (55.4)	.015
No	132 (56.4)	122 (53.0)	90 (44.6)	
**Number of health problems**				
<3	183 (78.2)	168 (73.0)	126 (62.4)	<.001
≥3	51 (21.8)	62 (27.0)	76 (37.6)	
**Charlson Comorbidity Index^g^**				
0–2 points	175 (74.8)	190 (82.6)	147 (72.8)	.70
≥3 points	59 (25.2)	40 (17.4)	55 (27.2)	
**Self-perceived health**				
Very poor, poor, or fair	42 (17.9)	44 (19.1)	84 (41.6)	<.001
Good or very good	192 (82.1)	186 (80.9)	118 (58.4)	
**Functional limitation^h^**				
Yes	51 (21.8)	43 (18.7)	58 (28.7)	.10
No	183 (78.2)	187 (81.3)	144 (71.3)	
**Locus of control^i^**				
Internal	160 (68.7)	162 (70.4)	115 (58.7)	.04
Other	73 (31.3)	68 (29.6)	81 (41.3)	
**Social support^j^**				
Weak	8 (3.5)	7 (3.1)	16 (8.1)	.03
Normal	223 (96.5)	221 (96.9)	182 (91.9)	

a Level of cancer worry was measured by the Spanish version of the CWS (2), made up of 6 questions with 4 response options (1 = not at all or rarely; 2 = sometimes; 3 = often; 4 = almost all the time), such that each individual attains a score ranging from 6 (minimum worry) to 24 (maximum worry).

b Through simple random sampling, participants residing in 8 basic health areas in the Province of Albacete in southwest Spain were selected on the basis of health card data, which reflect all people entitled to health care under Spain’s National Health System.

c Classes I and II = , higher-grade and lower-grade professionals, administrators and officials, managers, professions linked to university degrees; Classes III and IV = middle management and managerial staff, self-employed persons; Class V = , lower-grade skilled technicians; Classes VI and VII = semi-skilled and unskilled workers.

d Measured by International Physical Activity Questionnaire (12).

e Defined as consuming 280 g of alcohol per week (men) and 168 g per week (women).

f Assessed by a food frequency questionnaire (Cuestionario de Frecuencia de Consumo de Alimentos) (13) in accordance with guidelines of the Spanish Society of Community Nutrition (Sociedad Española de Nutrición Comunitaria).

g Charlson et al (14).

h Assessed with the EuroQol-5D questionnaire (15), which includes items on mobility, personal care, daily activities, pain or discomfort, and anxiety or depression.

i Locus of control refers to the extent to which people believe outcomes are determined by what they do (internal control) or are determined by events outside their control (external control); assessed using the 9-item Font scale (16).

j Assessed by the 11-item Duke–UNC Functional Social Support Questionnaire (17).

The mean CWS score was 9.3 points (SD, 3.1; 95% confidence interval [CI]: 9.0–9.5). Of those surveyed, 9.4% reported having often thought about the chance of developing cancer during the month preceding the interview, 3.5% reported that often thinking of cancer had affected their mood during the period, and 2.2% reported that having thoughts about their chances of getting cancer had affected their ability to perform daily activities. In addition, 31.9% of participants reported being worried about the possibility of developing cancer; 8.6% reported this as a frequent worry, and 22.5% said it was an important problem. CWS scores were significantly higher among women (9.7 [SD, 3.3]) than among men (8.7 [SD, 2.7]) (*t* = 4.17; *P* < .001), and scores had a weak negative correlation with age (Spearman ρ = −0.11; *P* = .003). For other sociodemographic characteristics, scores were significantly higher only among participants residing in rural areas (10.0 [SD, 3.4]) compared with those residing in urban areas (9.0 [SD, 3.0]) (*P* < .001).

ANOVA showed that cancer worry was greater among participants with a moderate or high level of physical activity (9.5 [SD, 3.2]) than among those with a low level of physical activity (8.9 [SD, 3.0]) (*P* = .01), among those with a personal history of cancer (10.8 [SD, 4.4] vs 9.1 [SD, 3.0]; *P* < .001) or family history of cancer (9.6 [SD, 3.2] vs 9.0 [SD, 3.0]; *P* = .008), among those who reported worse self-perceived health (very poor, poor, or fair) (10.6 [SD, 3.7] vs 8.8 [SD, 2.8]; *P* < .001), those with some functional limitation (10.0 [SD, 4.0] vs 9.1 [SD, 2.8]; *P* = .001), those having a locus of control other than internal (9.7 [SD, 3.3] vs 9.0 [SD, 3.0]; *P* = .008), and those with weak social support (10.3 [SD, 3.4] vs 9.2 [SD, 3.1]; *P* = .04). We found a weak positive correlation between CWS scores and the number of health problems reported by participants (Spearman ρ = 0.16; *P* < .001).

Multiple linear regression showed that 10 variables were independently associated with CWS scores (Table 4). The regression equation’s explanatory capacity was significant (*F* = 12.9; *P* < .001) and accounted for 16.7% (adjusted *R* ^2^ = 0.167) of the variability in the cancer worry value provided by the CWS. No correlation existed among the residual values (Durbin–Watson test statistic = 1.93). When the 58 participants with a personal history of cancer were excluded from the sensitivity analysis, the same variables were associated in the regression model. However, when the 322 participants with a family history of cancer were also excluded, only lower age and worse self-perceived health remained. Logistic regression showed that the variables associated with a CWS score of more than 10 (66th percentile) were female sex (odds ratio [OR], 1.59), personal history of cancer (OR, 2.30), family history of cancer (OR, 1.44), worse self-perceived health (OR, 2.74), locus of control other than internal (OR, 1.51), and rural setting (OR, 1.54).

**Table 4:** Variables Shown by Multiple Linear Regression to Be Associated With Cancer Worry Scale (CWS)^a^ Score in Study on Cancer Worry Among Adults Aged 50 or Older, Spain, 2012–2014^b^

Variable	Coefficient, B (95% Confidence Interval)	Standardized Coefficient	*t*	*P* Value
Constant	5.16 (2.25 to 8.06)	—	3.48	.001
Female sex	0.83 (0.38 to 1.29)	0.132	3.60	<.001
Lower age	−0.05 (−0.09 to −0.02)	−0.114	−3.06	.002
Rural setting	0.64 (0.14 to 1.14)	0.092	2.50	.01
Medium-to-high level of physical activity	0.89 (0.40 to 1.37)	0.136	3.57	<.001
Worse self-perceived health	1.15 (0.59 to 1.70)	0.160	4.05	<.001
Personal history of cancer	1.58 (0.78 to 2.38)	0.142	3.87	<.001
Family history of cancer	0.48 (0.04 to 0.93)	0.078	2.13	.03
Lower degree of social support	−0.03 (−0.06 to −0.001)	−0.077	−2.01	.04
Number of reported health problems	0.27 (0.10 to 0.43)	0.130	3.15	.002
Locus of control other than internal	0.56 (0.09 to 1.03)	0.085	2.33	.02

a Level of cancer worry was measured by the Spanish version of the CWS (2), made up of 6 questions with 4 response options (1 = not at all or rarely; 2 = sometimes; 3 = often; 4 = almost all the time), such that each individual attains a score ranging from 6 (minimum worry) to 24 (maximum worry).

b Through simple random sampling, participants residing in 8 basic health areas in the Province of Albacete in southwest Spain were selected on the basis of health card data, which reflect all people entitled to health care under Spain’s National Health System.

## Discussion

Although a low level of cancer worry was observed generally among study partcipants aged 50 or older, such worry was nevertheless reported by almost one-third of the study sample, and it posed an important problem to approximately one in 5 people. This worry was related to certain sociodemographic characteristics or lifestyles and was greater among younger people, women, those residing in a rural area, and those who engaged in higher levels of physical activity, a finding that should be confirmed by future studies, because evidence shows that such activity reduces the risk of various types of cancer (18). Increased cancer worry was also seen among participants who reported a personal or family history of cancer, more health problems, worse self-perceived health, or some functional limitation. Similarly, concern tended to be more pronounced among participants who reported a locus of control other than internal or weak social support.

This study has several limitations. Cancer worry was less pronounced in the presence of an interviewer than when participants answered a self-administered questionnaire, particularly among those who had a lower educational level. Surveys administered by an interviewer might thus underestimate cancer worry (19), which could have biased our results. Another limitation is that the study design did not enable causal relationships to be established between the variables and cancer worry. Furthermore, some self-selection bias might have occured if the study participation rate was lower among people with lower levels of cancer worry. Similarly, some information bias may have resulted from the participation of different interviewers, who may have influenced interviewees’ responses to differing degrees. Despite all the variables considered in the study, our results explain only part of the variability in cancer worry, which might have been overestimated by the stepwise procedure used to select the predictor variables.

In a study conducted in the United Kingdom (3), most of those interviewed (59%) reported fearing cancer more than any other disease and, similar to our findings, a considerable proportion of those surveyed (25%) expressed a marked degree of cancer worry, with such concern being higher among women. In addition, the study found a higher degree of worry among racial/ethnic minority populations and those with a lower educational level. Cancer worry is a complex, multifactorial phenomenon which, though still not well known or understood, should nevertheless be tackled in a health care setting to facilitate the adoption of preventive behavior. Perception of risk and emotions such as fear or worry can influence health protection motivations and actions, although no consensus exists on whether cancer worry motivates or inhibits cancer detection behaviors (20). Despite association with the regular performance of certain screening tests, such as mammography or colonoscopy, neither risk (susceptibility) nor worry is associated with determination of occult blood in the stool (21,22). The findings suggest that risk and worry are both important in the prediction of certain behavior patterns related to screening tests. Furthermore, perception of the risk of cancer, whether individually or comparatively with other people, is linked to cancer worry (23).

Health professionals should identify patients with excessive cancer worry and help them reduce this concern by providing them with information about appropriate preventive health care. To this end, it might be useful to ascertain which population groups feel more worried than other groups by this disease and which factors contribute significantly to such heightened concern. Once a person’s worry has been assessed, health professionals, through individualized counseling, can then help determine the person’s own risk according to his or her family history and respond to any concern. Moreover, knowing the circumstances that cause a high level of worry might enable a closer follow-up of the persons most affected and personalize educational interventions that would allay their worries. The CWS is an easily applicable instrument, and its adequate psychometric properties (2) mean that it can be used as a cancer worry assessment scale. It can also be a good instrument for comparing and ascertaining the factors that could modify the degree of cancer worry in different populations.

Outside the health care setting, the acquisition of knowledge about cancer through the news media might also be a suitable way of reducing worry about the disease, if the media is supplied reliable, unambiguous information about the prevention and detection of cancer (24), which would enable individuals to adopt the most appropriate measures. In our study, we found no relationship between cancer worry and social class. Even so, cancer worry has been described as differing according to socioeconomic level, possibly determined by the level of information supplied by news media such as television and the Internet. If the level of cancer worry is indeed lower among persons having a higher level of cancer knowledge, then education channeled through the news media could improve knowledge and so help reduce cancer worry. The greater worry observed among people living in rural settings may be related to lower levels of information about cancer in such settings, even though the incidence of some cancers is lower in rural areas. Today some differential characteristics are still seen in rural versus urban settings (25). For example, in rural areas, disease is accepted as an extrinsic natural evil, outside the scope of influence of any person’s behavior or lifestyle. Such attitudes might lead to greater noncompliance with preventive measures.

In contrast, people seeking information about cancer primarly through television and the Internet could have a more negative perception of health, and this perception might be associated with a greater fear of cancer. To help reduce the burden of cancer worry, educators and health professionals must be aware of the possible negative effects that the news media can sometimes have (26). Furthermore, the fear of receiving bad news about health can cause people to avoid searching for information that might well be crucial for maintaining health. In much the same way, cancer worry could be related to the avoidance of medical visits when people perceive a high probability of cancer (27).

As in earlier studies (28), ours shows that women have a greater degree of cancer worry than do men. This difference might reflect differences by sex in the acquisition of health information in general (29) and should be considered when designing intervention strategies to influence men and women’s perceptions of cancer risk and worry. Our results also indicate that a person’s own experience of cancer, at a personal or family level, can have an impact both on cancer worry and, as described elsewhere (30), on related behavior.

Future studies will have to confirm our results and ascertain the usefulness of different interventions, targeted both at reducing excessive worry and at increasing the participation of participants at risk in cancer prevention activities.

Although the level of cancer worry is low among people aged 50 or older, almost a third of people in this age group reported being worried about the possibility of developing cancer, and the level of such concern is related both to personal characteristics and to lifestyle and health status. More research is called for to better understand how contextual factors can strengthen or weaken health motivation. Advances in this area of research could enhance the effectiveness of interventions designed to change behavior patterns of cancer prevention activities, and in doing so, increase their potential public health benefit.

## References

[1] Jensen JD , Bernat JK , Davis LA , Yale R . Dispositional cancer worry: convergent, divergent, and predictive validity of existing scales. J Psychosoc Oncol 2010;28(5):470–89. 10.1080/07347332.2010.498459 20730660 PMC2928149

[2] Cabrera E , Zabalegui A , Blanco I . Versión española de la Cancer Worry Scale (Escala de Preocupación por el Cáncer: adaptación cultural y análisis de la validez y la fiabilidad). [Spanish version of the Cancer Worry Scale (CWS). Cross cultural adaptation and validity and reliability analysis]. Med Clin (Barc) 2011;136(1):8–12. 10.1016/j.medcli.2010.04.015 20561651

[3] Vrinten C , van Jaarsveld CH , Waller J , von Wagner C , Wardle J . The structure and demographic correlates of cancer fear. BMC Cancer 2014;14(1):597. 10.1186/1471-2407-14-597 25129323 PMC4148526

[4] Bowen DJ , Helmes A , Powers D , Anderson MR , Burke W , McTiernan A , Predicting breast cancer screening intentions and behavior with emotion and cognition. J Soc Clin Psychol 2003;22(2):213–32. 10.1521/jscp.22.2.213.22875

[5] Consedine NS , Magai C , Neugut AI . The contribution of emotional characteristics to breast cancer screening among women from six ethnic groups. Prev Med 2004;38(1):64–77. 10.1016/j.ypmed.2003.09.030 14672643

[6] Bernat JK , Jensen JD . Measuring dispositional cancer worry in China and Belgium: a cross-cultural validation. J Psychosoc Oncol 2014;32(2):189–206. 10.1080/07347332.2013.874002 24365045 PMC3997738

[7] Gramling R , Anthony D , Frierson G , Bowen D . The cancer worry chart: a single-item screening measure of worry about developing breast cancer. Psychooncology 2007;16(6):593–7. 10.1002/pon.1128 17096453

[8] Loescher LJ . Cancer worry in women with hereditary risk factors for breast cancer. Oncol Nurs Forum 2003;30(5):767–72. 10.1188/03.ONF.767-772 12949589

[9] Trask PC , Paterson AG , Wang C , Hayasaka S , Milliron KJ , Blumberg LR , Cancer-specific worry interference in women attending a breast and ovarian cancer risk evaluation program: impact on emotional distress and health functioning. Psychooncology 2001;10(5):349–60. 10.1002/pon.510 11536413

[10] Lerman C , Trock B , Rimer BK , Boyce A , Jepson C , Engstrom PF . Psychological and behavioral implications of abnormal mammograms. Ann Intern Med 1991;114(8):657–61. 10.7326/0003-4819-114-8-657 2003712

[11] Hopwood P , Shenton A , Lalloo F , Evans DG , Howell A . Risk perception and cancer worry: an exploratory study of the impact of genetic risk counselling in women with a family history of breast cancer. J Med Genet 2001;38(2):139. 10.1136/jmg.38.2.139 11288719 PMC1734804

[12] Craig CL , Marshall AL , Sjöström M , Bauman AE , Booth ML , Ainsworth BE , International physical activity questionnaire: 12-country reliability and validity. Med Sci Sports Exerc 2003;35(8):1381–95. 10.1249/01.MSS.0000078924.61453.FB 12900694

[13] Rodríguez IT , Ballart JF , Pastor GC , Jordà EB , Val VA . Validación de un cuestionario de frecuencia de consumo alimentario corto: reproducibilidad y validez. [Validation of a short questionnaire on frequency of dietary intake: reproducibility and validity]. Nutr Hosp 2008;23(3):242–52. 18560701

[14] Charlson ME , Pompei P , Ales KL , MacKenzie CR . A new method of classifying prognostic comorbidity in longitudinal studies: development and validation. J Chronic Dis 1987;40(5):373–83. 10.1016/0021-9681(87)90171-8 3558716

[15] EuroQol Group. EuroQol — a new facility for the measurement of health-related quality of life. Health Policy 1990;16(3):199–208. 10.1016/0168-8510(90)90421-9 10109801

[16] Font A . Locus de control en situaciones de indefensión producida por amenaza real. Psicologemas 1989;3:225–43.

[17] Broadhead WE , Gehlbach SH , de Gruy FV , Kaplan BH . The Duke–UNC Functional Social Support Questionnaire. Measurement of social support in family medicine patients. Med Care 1988;26(7):709–23. 10.1097/00005650-198807000-00006 3393031

[18] Anzuini F , Battistella A , Izzotti A . Physical activity and cancer prevention: a review of current evidence and biological mechanisms. J Prev Med Hyg 2011;52(4):174–80. 22442921

[19] Persoskie A , Leyva B , Ferrer RA . Mode effects in assessing cancer worry and risk perceptions: is social desirability bias at play? Med Decis Making 2014;34(5):583–9. 10.1177/0272989X14527173 24718657 PMC4192106

[20] Hay JL , Buckley TR , Ostroff JS . The role of cancer worry in cancer screening: a theoretical and empirical review of the literature. Psychooncology 2005;14(7):517–34. 10.1002/pon.864 15490428

[21] Hay JL , McCaul KD , Magnan RE . Does worry about breast cancer predict screening behaviors? A meta-analysis of the prospective evidence. Prev Med 2006;42(6):401–8. 10.1016/j.ypmed.2006.03.002 16626796

[22] Moser RP , McCaul K , Peters E , Nelson W , Marcus SE . Associations of perceived risk and worry with cancer health-protective actions: data from the Health Information National Trends Survey (HINTS). J Health Psychol 2007;12(1):53–65. 10.1177/1359105307071735 17158840

[23] Zajac LE , Klein WM , McCaul KD . Absolute and comparative risk perceptions as predictors of cancer worry: moderating effects of gender and psychological distress. J Health Commun 2006;11(Suppl 1):37–49. 10.1080/10810730600637301 16641073

[24] Han PK , Moser RP , Klein WM . Perceived ambiguity about cancer prevention recommendations: relationship to perceptions of cancer preventability, risk, and worry. J Health Commun 2006;11(Suppl 1):51–69. 10.1080/10810730600637541 16641074 PMC4194067

[25] [Rural medicine: a view to the future (and II). Rural Medicine Working Group of the semFYC]. Aten Primaria 2000;26(3):187–93. 10996955 10.1016/S0212-6567(00)78640-9 PMC7683947

[26] Nelissen S , Beullens K , Lemal M , Van den Bulck J . Predictors of cancer fear: the association between mass media and fear of cancer among cancer diagnosed and nondiagnosed individuals. J Cancer Educ 2015;30(1):68–74. 10.1007/s13187-014-0705-z 25270554

[27] Persoskie A , Ferrer RA , Klein WM . Association of cancer worry and perceived risk with doctor avoidance: an analysis of information avoidance in a nationally representative US sample. J Behav Med 2014;37(5):977–87. 10.1007/s10865-013-9537-2 24072430

[28] McQueen A , Vernon SW , Meissner HI , Rakowski W . Risk perceptions and worry about cancer: does gender make a difference? J Health Commun 2008;13(1):56–79. 10.1080/10810730701807076 18307136

[29] Gonzalez JS , Chapman GB , Leventhal H . Gender differences in the factors that affect self-assessments of health. J Appl Biobehav Res 2002;7(2):133–55. 10.1111/j.1751-9861.2002.tb00081.x

[30] Benyamini Y , McClain CS , Leventhal EA , Leventhal H . Living with the worry of cancer: health perceptions and behaviors of elderly people with self, vicarious, or no history of cancer. Psychooncology 2003;12(2):161–72. 10.1002/pon.637 12619148

